# A single bout of moderate-intensity aerobic exercise improves motor learning in premanifest and early Huntington’s disease

**DOI:** 10.3389/fpsyg.2023.1089333

**Published:** 2023-03-08

**Authors:** Sophie C. Andrews, Lydia Kämpf, Dylan Curtin, Mark Hinder, Nicole Wenderoth, Julie C. Stout, James P. Coxon

**Affiliations:** ^1^School of Psychological Sciences, Turner Institute for Brain and Mental Health, Monash University, Clayton, VIC, Australia; ^2^Healthy Brain Ageing Research Group, Thompson Institute, University of the Sunshine Coast, Birtinya, QLD, Australia; ^3^Neural Control of Movement Lab, Department of Health Sciences and Technology, ETH Zürich, Zurich, Switzerland; ^4^Sensorimotor Neuroscience and Ageing Research Group, School of Psychological Sciences, College of Health and Medicine, University of Tasmania, Hobart, TAS, Australia; ^5^Neuroscience Center Zurich (ZNZ), Federal Institute of Technology Zurich, University and Balgrist Hospital Zurich, University of Zurich, Zurich, Switzerland; ^6^Future Health Technologies, Singapore-ETH Centre, Campus for Research Excellence and Technological Enterprise (CREATE), Singapore, Singapore

**Keywords:** motor skills, implicit learning, cardiovascular exercise, premanifest Huntington’s disease, neuroplasticity

## Abstract

**Introduction:**

Cardiorespiratory exercise has emerged as a promising candidate to modify disease progression in Huntington’s disease (HD). In animal models, exercise has been found to alter biomarkers of neuroplasticity and delay evidence of disease, and some interventions–including exercise–have shown benefits in human HD patients. In healthy human populations, increasing evidence suggests that even a single bout of exercise can improve motor learning. In this pilot study, we investigated the effect of a single bout of moderate intensity aerobic exercise on motor skill learning in presymptomatic and early manifest HD patients.

**Methods:**

Participants were allocated to either an exercise (*n* = 10) or control (*n* = 10) group. They performed either 20 min of moderate intensity cycling or rest before practicing a novel motor task, the sequential visual isometric pinch force task (SVIPT). After 1 week, the retention of the SVIPT was measured in both groups.

**Results:**

We found that the exercise group performed significantly better during initial task acquisition. There were no significant differences in offline memory consolidation between groups, but total skill gain across both acquisition and retention sessions was greater in the group who exercised. The better performance of the exercise group was driven by improvements in accuracy, rather than speed.

**Discussion:**

We have shown that a single bout of moderate intensity aerobic exercise can facilitate motor skill learning in people with HD gene-expansion. More research is needed to investigate the underlying neural mechanisms and to further explore the potential for neurocognitive and functional benefits of exercise for people with HD.

## 1. Introduction

Huntington’s disease (HD) is an autosomal dominant neurodegenerative disease, and affects movement, cognition, and emotion (Walker, 2007). While HD usually first appears in midlife, age of onset is highly variable and appears to be influenced by a variety of genetic and environmental influences, including aspects of lifestyle (Mo et al., 2015). The potential for physical activity to benefit the brain and cognitive performance in people with the genetic expansion for HD was first explored in HD mouse-model studies. These studies showed that voluntary wheel running increased biomarkers of neuroplasticity, such as brain-derived neurotrophic factor (BDNF; Pang et al., 2006; Zajac et al., 2010), and delayed disease progression, including cognitive and motor signs (Harrison et al., 2013; Stefanko et al., 2017; Caldwell et al., 2020). Recently, exercise interventions have been trialed in people with HD with mixed results (Fritz et al., 2017; Playle et al., 2019), although one pilot study of a multidisciplinary intervention that included exercise increased gray matter and improved learning and memory over 9 months (Cruickshank et al., 2015; Bartlett et al., 2020). One recent study revealed the acute neuroplasticity response to both moderate- and high-intensity exercise (measured using transcranial magnetic stimulation, TMS) was reduced in premanifest and early HD compared to controls without the gene-expansion (Andrews et al., 2022). This demonstrates the importance of investigating effects of exercise on cognition in HD, and exploring potential underlying mechanisms, as these may differ from the general population. In people with the expanded HD gene, the acute effects of exercise on learning and memory have not been studied.

One key type of learning and memory, which is affected early in HD, is motor skill learning. Motor learning is the process that underpins the acquisition and retention of motor skills, such as writing or riding a bicycle, and is important for independent living. Learning to perform such tasks often incurs a speed-accuracy trade-off (Fitts’ law), whereby increased movement speed comes at the cost of reduced accuracy, or vice versa (Dayan and Cohen, 2011). However, with repetitive practice, movements can be executed faster and more accurately to achieve a goal, reflecting the development of skill (Willingham, 1998; Dayan and Cohen, 2011). People with HD consistently show impairments in motor skill learning, even prior to motor onset, particularly in sequence learning (Heindel et al., 1988; Willingham and Koroshetz, 1993; Willingham et al., 1996; Smith et al., 2000; Smith and Shadmehr, 2005). A task well-suited to quantifying the development of motor skill is the sequential visuomotor isometric pinch force task (SVIPT), as it allows participants to vary their emphasis on speed or accuracy (Reis et al., 2009, 2015; Statton et al., 2015; Stavrinos and Coxon, 2017), but this task has not yet been utilized to investigate motor skill learning in HD. It shares similarities with previous motor learning tasks utilized in HD populations in that it involves sequence learning (Willingham and Koroshetz, 1993; Holtbernd et al., 2016). The task is tailored to a participant’s maximum voluntary contraction, and has previously been successfully adapted for use in a clinical population (Hardwick et al., 2017), making it a suitable choice for use with an HD population.

Improvements in motor skills can occur during training (online skill acquisition), and also after training (offline motor memory consolidation), where new memories are transformed from their initial fragile states into more robust forms, most likely *via* long-term potentiation (LTP) like changes of synaptic strength (Butefisch et al., 2000; Rosenkranz et al., 2007). Previous studies investigating the effects of exercise on motor learning in healthy samples have found effects from a single bout. Studies of healthy young adults have indicated benefits of an acute bout of high-intensity exercise on both motor skill acquisition (Mang et al., 2014) and consolidation (Roig et al., 2012; Skriver et al., 2014; Stavrinos and Coxon, 2017), [although not all, see for example: Pixa et al. (2021), Quinlan et al. (2021)]. In some healthy young adult studies, moderate-intensity exercise has also been shown to facilitate motor learning. For example, 30 min of treadmill running resulted in improved motor skill acquisition of a SVIPT conducted immediately afterward, but not in subsequent retention; this improvement was driven by changes in accuracy, rather than speed (Statton et al., 2015). Furthermore, Moriarty et al. (2022) found improved motor learning *via* a piano task following moderate-intensity exercise but not high-intensity exercise. Whether an acute bout of exercise affects motor skill acquisition, retention, or both in HD, is unknown.

In HD, neurocognitive deficits such as motor learning commonly precede the onset of motor symptoms (Stout et al., 2012; Holtbernd et al., 2016), although there is individual variation in the order of symptom onset and progression (McAllister et al., 2021). The aim of the current study was therefore to investigate whether an acute bout of moderate-intensity exercise would improve motor learning (both skill acquisition and retention) in people with presymptomatic and early manifest HD, measured using a SVIPT task. HD participants were randomly allocated to either an exercise or a control group. We hypothesized that participants in the exercise group would show increased acquisition and retention compared to the control group.

## 2. Materials and methods

### 2.1. Participants

Twenty one right-handed people with premanifest or early motor manifest HD were recruited, and 20 completed this study (11 women, mean age = 50.14 ± 12.18 years, range = 27–70). Participants were recruited from the Experimental Neuropsychology Research Unit (ENRU-Stout) participant database held at Monash University (Melbourne), the Statewide Progressive Neurological Disease Service at Calvary Healthcare Bethlehem (Melbourne), and the Tasmanian Health Service (Hobart and Launceston). The study was conducted at Monash University, Melbourne, and University of Tasmania, Hobart and Launceston. Participants were required to have a genetically confirmed expansion of the HD CAG repeat sequence (≥ 39 CAG repeats), and no more than mild-moderate functional impairment, defined as a Total Functional Capacity (TFC) score of ≥ 9 on the Unified Huntington’s disease Rating Scale (Huntington Study Group, 1996). To limit the study to participants within an estimated 15 years of onset (relatively near to onset), we included only people with a Disease Burden Score [DBS; calculated as age × (CAG-35.5)] of > 200 (Penney et al., 1997). Exclusion criteria included significant medical or neurological condition (other than HD), traumatic brain injury, cardiovascular risk factors, or severe psychiatric illness including substance dependence. Participants with mild to moderate symptoms of anxiety or depression were not excluded. In addition, color-blind individuals were excluded, due to the requirements of the motor task. Participants were screened for contraindications to exercise with the Adult Pre-Exercise Screening System (APSS; Sports Medicine Australia, 2011).

Participants were classified as premanifest if they had never received a clinical diagnosis of HD, and as early HD if they had received a clinical diagnosis of HD. For participants who also participated in the longitudinal, observational Enroll-HD study (Sathe et al., 2021; *n* = 10), UHDRS Total Motor Score (TMS) was obtained from their most recent annual visit. Anxiety and depression symptoms were assessed using the Hospital Anxiety and Depression Scale (HADS; Zigmond and Snaith, 1983), and current physical activity levels were assessed using the International Physical Activity Questionnaire (IPAQ; Craig et al., 2003). Written informed consent was obtained before commencement of the study. Ethical approval was granted by the Monash University Human Research Ethics Committee, and the Research Integrity and Ethics Unit, University of Tasmania. All procedures were undertaken in accordance with the World Medical Association Declaration of Helsinki.

### 2.2. Study design

**FIGURE 1:**
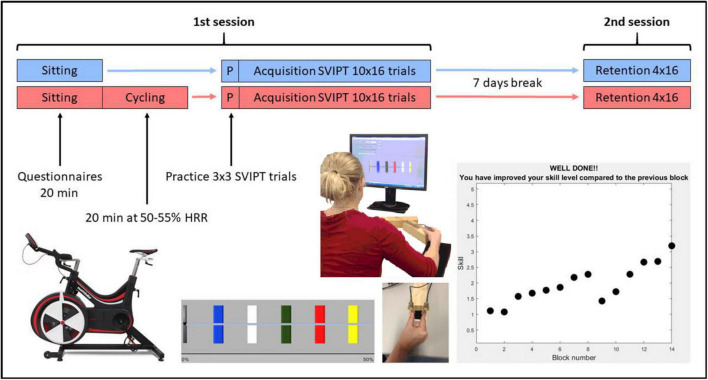
Summary of the study protocol with the exercise group in red and the control group in blue. SVIPT, sequential visual isometric pinch task; HRR, heart rate reserve.

Participants were pseudorandomized to either an exercise group (*n* = 10) or a control group (*n* = 10) while minimizing variance in gender, age, DBS and TFC using an online algorithm.^1^ A single randomization list was used across both sites. As shown in Figure 1, the experiment consisted of two sessions. Participants were asked to refrain from strenuous physical activity for the 24 h leading up to each of the sessions. In the first session, participants were fitted with a chest-strap heart-rate monitor (Polar H7, Polar Electro), then were seated and completed questionnaires for 20 min, while their resting heart rate was recorded. Following this, they either undertook 20 min of moderate-intensity aerobic exercise on a stationary bike (exercise group; see below for details), or sat quietly on a chair (control group) for an equivalent period of time. After a 10 min recovery period, participants undertook a motor learning task lasting ∼40 min, which is described below. One week later (± 1 day), participants returned and retention of the motor skill was assessed. Learning and retention sessions were scheduled at the same time of day for each participant, where possible (60% of participants, with no differences in distribution between the two groups).

### 2.3. Exercise protocol

The exercise was performed on a stationary cycle ergometer (Wattbike, Geelong Australia; or Enduro Infiniti CB2110, NSW, Australia). Moderate intensity exercise was performed for 20 min after a 3–5 min warm-up (at low intensity). This intensity and length of exercise bout was selected due to our older, clinical population, in order to reduce risk of fatigue. Moderate intensity exercise was defined as 50–55% of heart rate reserve (HRR; Norton et al., 2010), which was calculated by subtracting resting heart rate from their age-predicted maximum heart rate (220-current age). For the exercise group, in addition to monitoring of heart rate throughout the session, participants’ ratings of exercise intensity were also obtained every 5 min using Borg’s perceived exertion scale, which is a self-reported measure ranging from 6 (no exertion at all) to 20 (maximal exertion) (Borg, 1970).

### 2.4. SVIPT task

LabView and MATLAB were used to implement the motor learning task using custom software and scripts. All participants were naive to the SVIPT. Participants were seated approximately 60 cm in front of a computer screen while holding a custom in-house force transducer between the index finger and thumb of their dominant right hand on the table in front of them (Figure 1). Squeezing the transducer moved a cursor on the screen horizontally. For each trial, the goal was to move the cursor into five colored targets following a specific sequence (red–blue–green–yellow–white) and return to the home position after each pulse by relaxing. The order of the colored targets was the same for all trials. Participants were instructed to start as soon as the colors appeared, land the peak of their force pulses in the center of the targets, and try to perform the sequence as quickly and accurately as possible. The furthest target was set at 45% of the participant’s maximum voluntary contraction (MVC), and the width of each target was 2.5% of their MVC. MVC was determined from the participant’s maximal brief pinch on the force transducer, with the force pulse lasting approximately 0.5 s. A logarithmic transformation was applied to the relationship between force and cursor movement to increase task difficulty. Participants were not informed of this transformation, and had to learn the relationship implicitly while they performed the task.

To ensure that participants were familiar with the requirements of the pinch task, they completed 3 mini-blocks of 3 practice trials before commencing the learning blocks. The main learning task comprised 10 blocks of 16 trials each (40–50 min total duration). In the retention session 1 week later, they completed a further 4 blocks of 16 trials each (15–20 min total duration). As motor learning is sensitive to both positive and negative feedback (Galea et al., 2015), visual feedback of their performance (a combination of accuracy and speed) was given at the end of each block (Figure 1). One of two possible messages were displayed: (1) “Well done! You have improved your skill level compared to the previous block.”; or (2) “Keep trying! Your skill level decreased compared to the previous block.” There was approximately 5 s between each trial and a 1–2 min break between blocks while feedback was displayed.

### 2.5. Data processing and analysis

Each SVIPT trial was examined and excluded from further analysis if it failed to meet specific criteria (for a full description of these criteria and graphical examples, see the Supplementary material). The mean percentage of excluded trials per participant was 8.5 (SD = 7.5) (for group comparison see Table 1). After exclusion of single trials, outliers more than 2.5 standard deviations above or below the mean were replaced by the next closest data point in the respective block (windsorizing; Wilcox, 2017).

**TABLE 1:** Participant means and standard deviations for the control and exercise groups.

	Control	Exercise	*p*-value
n (men)	10 (4)	10 (5)	0.65
Age (years)	53.7 (10.3)	48.4 (13)	0.33
CAG repeat number	42.2 (1.9)	43.4 (2.8)	0.27
Disease Burden Score (DBS)	351.3 (84.8)	359.4 (80.6)	0.83
N presymptomatic HD	7	4	0.18
TFC	12 (1.5)	12 (1.6)	0.67
TMS	5 (6)^a^	6 (4)^a^	0.87
HADS: Anxiety	3.7 (2.8)	6 (3.1)	0.10
HADS: Depression	2.4 (3.1)	4.5 (3.3)	0.16
IPAQ MET-min/week	9290 (9196)^b^	9929 (7368)	0.87
RHR (bpm)	68.67 (6.3)^c^	68 (9.7)	0.90
HRR (bpm)		103.5 (15.2)	−
HR (moderate intensity)		123 (11.6)	−
RPE		12.6 (0.6)	−
Excluded trials (% of total)	8 (8.1)	9 (7.2)	0.77

TFC, Total Functional Capacity; TMS, Total Motor Score; IPAQ, International Physical Activity Questionnaire; HADS, Hospital Anxiety and Depression Scale; HRR, heart rate reserve; RPE, Received Perception of Exertion (Borg scale).

^a^TMS only available for 5 participants in each group.

^b^IPAQ available for *n* = 9.

^c^RHR only available for 5 participants in the control group.

Data analysis was carried out using MATLAB R2018b and IBM SPSS 25. Frequentist analyses were undertaken, with the *a priori* alpha level set to 0.05. The main outcome measure for the performance on the SVIPT was a skill measure which was derived for each trial from the total time to complete the trial (speed) and total force error of the force peaks (accuracy) (Stavrinos and Coxon, 2017). Total trial time was calculated from appearance of the colored targets to the completion of the last detected force pulse. Trial force error was calculated as the sum of differences between the center of each target and the participant’s five respective force peaks. In order to estimate an overall skill level, we used a previously determined function [for further detail see Stavrinos and Coxon (2017)]:


(1) $$\mathrm{Skill}\,\mathrm{parameter}=\frac{1-\mathrm{force}\,\mathrm{error}}{\mathrm{force}\,\mathrm{error}*\left(\text{log}\,{\left(\mathrm{duration}\right)}^{1.627}\right)}$$

where “duration” refers to the total time to complete the trial, and larger values of the skill parameter reflect greater performance. To reduce heteroscedasticity, the skill parameter was log transformed resulting in the final skill measure (Reis et al., 2009). To account for individual differences in baseline motor performance, this skill measure was standardized to the relevant group mean Block 1 performance, so that the relative learning trajectories could be assessed (Reis et al., 2015; Kolasinski et al., 2019).

The Shapiro-Wilk test was used to assess the assumption of normality for each dependent variable, for each level of the independent variable, and violations were infrequent (< 10%) and were not systematic. Baseline difference between control and exercise groups was assessed using an independent-samples *t*-test. A series of two-way mixed model ANOVAs with Group (exercise and control) as between-subjects factor and performance on the relevant Blocks as within-subjects factor, was used to compare skill across the groups during acquisition (online effects; Blocks 1–10), following the 7-days break (offline effects; Block 10 vs. 12), and across both sessions (total effects; Blocks 1–14). Block 12 was used rather than Block 11, to allow for participants to refamiliarize themselves with the task after the 7-days break, i.e., to account for the known warm-up decrement (Reis et al., 2015). Independent measures *t*-tests compared the exercise and control groups on their online, offline and total effects for the skill measure. Further, to investigate whether any effects could be attributed to speed or accuracy, the ANOVAs and *t*-tests were also conducted on the raw data used to calculate the skill measure. When the assumption of sphericity was violated (Epsilon > 0.7), a Huynh-Feldt correction was applied.

## 3. Results

### 3.1. Group characteristics

There were no statistically significant differences between groups for age, gender, disease severity scores (i.e., DBS, TFC, TMS, HADS scores), self-reported physical activity levels (IPAQ), resting heart rate, maximum voluntary contraction, or percentage of excluded trials, all *ps* > 0.10 (see Table 1). 11 participants were prescribed antidepressant medications (4 × SSRI, 1 × SNRI, 1 × Tetracyclic, 5 × unspecified), 2 were prescribed migraine medications, 1 was taking hormone replacement therapy, and 1 was prescribed medication for the management of diabetes.

### 3.2. Baseline performance and skill acquisition

There were no significant differences in skill between groups at baseline [Block 1: exercise group *M* = 0.80, SD = 0.20; control group *M* = 0.96, SD = 0.32; *t*(1,14.85) = 1.29, *p* = 0.22]. There were also no group differences at baseline on accuracy [*t*(1,18) = 0.92, *p* = 0.37] or speed [*t*(1,18) = 0.04, *p* = 0.97].

### 3.3. Skill acquisition

**FIGURE 2:**
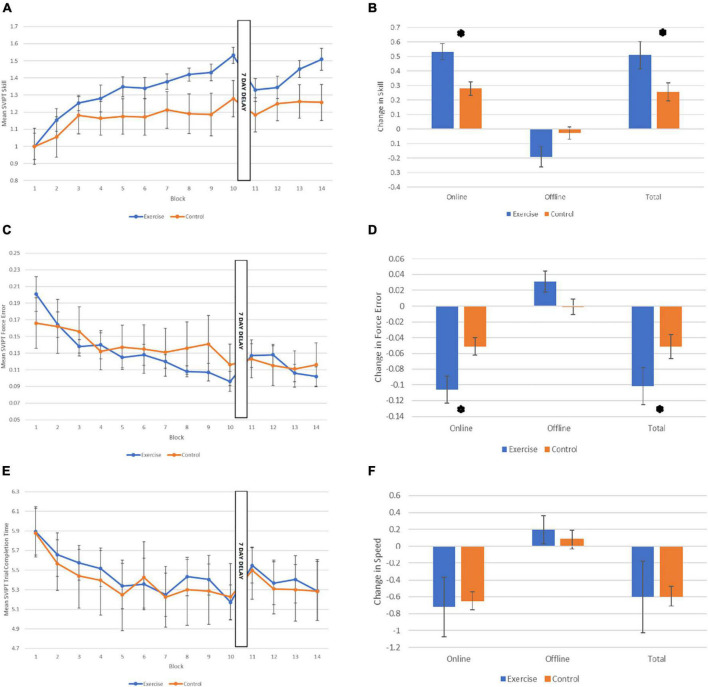
Performance on the motor learning task across the two sessions. **(A)** Mean sequential visual isometric pinch task (SVIPT) skill score for each group for each of the 14 Blocks. **(B)** Skill change scores for each group: Online (from Block 1 to 10), offline (from Block 10 to 12), and total change in skill (from Block 1 to 14; normalized to Block 1). **(C)** Mean SVIPT force error (accuracy) score for each group for each of the 14 Blocks. **(D)** Force error change scores for each group: Online, offline, and total change in force error. **(E)** Mean SVIPT trial completion time (in secs) for each group for each of the 14 Blocks. **(F)** Speed change scores for each group: Online, offline, and total change in speed. All data are presented as M (SE). *p < 0.05.

With regard to online skill acquisition (across Session 1 blocks), as shown in Figure 2A, there was a main effect of Block, with performance improving over time across both groups (*F*_7.3,132_ = 21.94, *p* < 0.001, η_p_^2^ = 0.55). There was also a significant Block x Group interaction effect, with the exercise group exhibiting more improvement in performance across blocks than the control group (*F*_7.3,132_ = 2.83, *p* = 0.008, η_p_^2^ = 0.14). In contrast, the main effect of Group was not statistically significant, (*F*_1,18_ = 1.75, *p* = 0.20, η_p_^2^ = 0.88). Independent samples *t*-tests revealed that the exercise group demonstrated significantly more skill gain during the acquisition phase, and in total across both learning and retention sessions, in comparison to the control group (see Figure 2B; Online Skill Change: *t*_1,18_ = 3.49, *p* = 0.003, *d* = 1.56). When examining total skill change across both sessions (Block 1–Block 14) significantly improved, represented as a significant main effect of Block (*F*_8.15,146.7_ = 15.40, *p* < 0.001, η_p_^2^ = 0.46). There was no significant main effect of Group (*F*_1,18_ = 2.01, *p* = 0.17, η_p_^2^ = 0.10), and no significant interaction effect (*F*_8.15,146.7_ = 1.95, *p* = 0.056, η_p_^2^ = 0.10), although, the total skill gain from Block 1 to 14 was significantly higher in the exercise group compared to the control group [Figure 2B: *t*(1,18) = −2.70, *p* = 0.037, *d* = 1.01]. There was no difference in performance between exercise and control groups with regard to offline effects on skill. Specifically, participants in both groups demonstrated a drop in performance from Block 10 to Block 12, reflected by a main effect of Time (*F*_1,18_ = 7.01, *p* = 0.016, η_p_^2^ = 0.28). Neither the main effect of Group (*F*_1,18_ = 2.49, *p* = 0.13, η_p_^2^ = 0.12), and nor interaction between Time and Group (*F*_1,18_ = 3.98, *p* = 0.061, η_p_^2^ = 0.18) were statistically significant.

### 3.4. Accuracy

When assessing accuracy and speed performances separately, it was apparent that the advantage seen by the exercise group during acquisition was driven by greater reductions in force error i.e., improved accuracy, rather than improvements in speed. Specifically, the exercise group showed significantly reduced force error across blocks than the control group (Figure 2C; Block × Group interaction: *F*_6.99,125.81_ = 2.52, *p* = 0.019, η_p_^2^ = 0.12), and this was reflected in a significantly reduced overall force error in the exercise group compared to the control group [Figure 2D; *t*(1,18) = −2.7, *p* = 0.014, *d* = 0.1.21]. There was also a significant main effect of Block (*F*_6.99,125.81_ = 12.93, *p* < 0.001, η_p_^2^ = 0.42), but the main effect of Group was not statistically significant (*F*_1,18_ = 0.08, *p* = 0.78, η_p_^2^ = 0.004).

### 3.5. Speed

As shown in Figure 2E, both groups improved their speed across blocks, reducing their mean time taken to complete the trials, and there were no significant differences in speed between groups, reflected by a significant main effect of Block (*F*_3.86,69.50_ = 5.75, *p* < 0.001, η_p_^2^ = 0.24), but no main effect of Group (*F*_1,18_ = 0.03, *p* = 0.86, η_p_^2^ = 0.002), or interaction between Block and Group (*F*_3.86,69.50_ = 0.20, *p* = 0.93, η_p_^2^ = 0.01). There were also no differences in speed between groups during the acquisition stage, [Figure 2F; *t*(1,10.64) = 0.42, *p* = 0.84, *d* = 0.09].

## 4. Discussion

This is the first study to investigate the acute effects of exercise on motor learning in people with the HD gene-expansion. Our finding that skill acquisition improved following moderate-intensity exercise, in comparison to rest, builds on the findings of Statton and colleagues, who also reported an improvement in the acquisition of the SVIPT task immediately after an acute bout of 30 min moderate-intensity running exercise in a healthy adult population (Statton et al., 2015). Similar to the current study, skill improvements were also driven by improvements in accuracy, rather than speed. This makes sense given that motor learning impairments previously observed in premanifest HD (using a field adaption paradigm) were driven by poor accuracy, and specifically a dysfunction in online error feedback control (Smith et al., 2000; Smith and Shadmehr, 2005). Therefore, it is possible that improvements in accuracy seen in the current study may reflect an improvement in on-line error correction during the SVIPT. Importantly, we also found an improvement in total skill gain over the two sessions, indicating that the skill gained during the acquisition phase was successfully consolidated and retrieved during the retention session 1 week later.

What potential neural mechanisms might drive this improved learning when preceded by exercise? TMS studies in healthy adults have shown that a single bout of 20–30 min cycling at moderate intensity can increase facilitation involving glutamate (Singh et al., 2014) and reduce GABA-mediated inhibition (Smith et al., 2014; Mooney et al., 2016) in the primary motor cortex, which indicates that a single bout of moderate intensity exercise may prime LTP-(like) neuroplasticity to promote motor learning. However, our recent TMS study of M1 synaptic plasticity following exercise in premanifest and early HD, however, revealed attenuated cortico-motor excitability, GABA-ergic short-interval cortical inhibition and glutamatergic facilitation responses following both high-intensity interval and moderate-intensity continuous cycling, indicating that these mechanisms are altered in HD (Andrews et al., 2022). Given that previous studies in presymptomatic HD participants have found different brain activation patterns during motor learning compared to adults without the HD gene expansion (Feigin et al., 2006; Holtbernd et al., 2016), one possibility is that exercise might help to promote neuroplasticity in these other regions to compensate for neurodegeneration in motor learning-related brain areas and boost compensatory networks. This is encouraging, as it suggests that even if M1 plasticity mechanisms are attenuated in HD, exercise may still have a beneficial effect on motor learning. This finding may therefore provide the first evidence that the benefit of exercise to brain and cognitive function, previously seen in HD mouse-models, may also apply to people with the HD gene-expansion.

Another potential explanation for improved performance during the acquisition phase, immediately following exercise, could partially be explained by increased arousal and motivation, as acute exercise is suggested to increase mood and attention as well as cognitive performance (Lambourne and Tomporowski, 2010; Smith et al., 2010). This is unlikely to account for the performance in its entirety, however, because overall skill gain from the first block to the last block across the two sessions was also significantly larger in the exercise group. This indicates a lasting change in skill, reflective of learning processes, rather than temporary improvement in performance due to arousal or motivation. Nevertheless, future research should investigate the relative roles of psychological and learning mechanisms in any improvement following exercise in motor skill.

The absence of a significant offline effect in our study is also consistent with Statton et al. (2015), who found no between-day retention of the SVIPT task when moderate-intensity exercise (defined as 65–85% age-predicted HRmax) was performed before the acquisition. In contrast with our findings, 20 min of high-intensity interval cycling was found to improve retention of a motor learning task (Roig et al., 2012; Stavrinos and Coxon, 2017). Indeed, the release of neuromodulators that can upregulate or downregulate neuroplasticity in response to exercise, and drive consolidation, seem to be intensity dependent (Knaepen et al., 2010; McDonnell et al., 2013; Skriver et al., 2014). This observation may explain the difference between our study and those utilizing high-intensity protocols. A possible mechanism for the effect of cardiovascular exercise on motor skill retention are increases in synthesis and release of BDNF which is crucial for synaptic plasticity, with the highest BDNF levels being detected after high-intensity protocols (Lipsky and Marini, 2007; Saucedo Marquez et al., 2015). Also, higher concentrations of norepinephrine after high-intensity exercise were associated with better retention after 7 days, and increased lactate levels with better retention 1 h as well as 24 h and 7 days after practice of a visuomotor tracking task (Skriver et al., 2014). Taken together, these findings indicate that moderate intensity exercise may not release sufficient amounts of neurotrophins and catecholamines for an offline learning effect in our study. Alternatively, given the improvement seen in the exercise group during acquisition, another possible explanation is that the exercise group reached a ceiling of improvement on the SVIPT task, which resulted in reduced opportunity for offline consolidation effects over and above that seen during acquisition. However, ceiling effects are unlikely because the SVIPT has previously been used in multi-day training studies (Reis et al., 2009, 2015; Quinlan et al., 2021).

With regard to limitations, because this was a pilot study and this paradigm has not been used before in an HD population, we did not undertake a sample size calculation. Sample size is comparable to other studies utilizing this motor skill learning paradigm to assess effects of exercise [e.g., (Statton et al., 2015; Stavrinos and Coxon, 2017)], however, we may have been underpowered to detect some effects. However, our study provides important, preliminary data regarding exercise and motor learning in HD, which can be utilized in the design of future, larger studies further exploring potential benefits in this population. Another limitation is that we did not assess general cognitive function in the participants, and so could not match the exercise and control groups on this variable specifically. Also, because the exercise/rest intervention was applied prior to the motor skill learning, this did not allow us to compare groups on baseline motor skill without any effects of exercise. Additionally, we did not include graded exercise testing to better tailor exercise intensity to individual fitness levels. These should be considered for future study designs. Although the performance improvements observed in the exercise group indicate an exercise-induced boost to learning, the absence of any neurophysiological measures of brain activity mean that any conclusions about underlying mechanisms is purely speculation at this point. Future research should combine both neurophysiological and motor learning measures into the one study, to better understand the relationships between these brain and behavior in response to exercise in HD. Additionally, although we have demonstrated the potential for an acute bout of exercise to improve motor learning, the effects of longer-term exercise interventions on people with the HD gene-expansion, and its potential to delay disease progression, remains unknown.

In conclusion, these results provide the first direct evidence for benefits to motor learning from a single bout of moderate-intensity exercise in premanifest and early HD. If replicated in a larger sample, this is a crucial step in bridging the gap between mechanistic work undertaken in HD mouse models on the benefit of exercise to the brain in HD, and the successful implementation of exercise interventions in people with the HD gene-expansion. Future research should investigate the effects of exercise on motor learning over repeated sessions, and include neurophysiological measures to elucidate the underlying mechanisms, so this can inform the design of future HD interventions to boost brain health, improve function and potentially delay disease progression in this population.

## Data availability statement

The raw data supporting the conclusions of this article will be made available by the authors, without undue reservation.

## Ethics statement

The studies involving human participants were reviewed and approved by Monash University Human Research Ethics Committee; Research Integrity and Ethics Unit, University of Tasmania. The patients/participants provided their written informed consent to participate in this study.

## Author contributions

SA, JS, and JC conceptualized and designed the study. SA, DC, and LK acquired the data. LK and JC processed the data. LK and SA ran the analyses and drafted the manuscript. All authors contributed to the interpretation of the data and reviewed and edited the manuscript.

## Footnotes

1
www.rando.la
