# Supplementary material for: A single bout of moderate-intensity aerobic exercise improves motor learning in premanifest and early Huntington’s disease
Source: Front Psychol. 2023 Mar 8;14:1089333. doi: 10.3389/fpsyg.2023.1089333 (PMC10032374; doi:10.3389/fpsyg.2023.1089333)
Supplement: Supplementary file 1 [file Data_Sheet_1.docx]

Supplementary Material

# SVIPT Data Cleaning

SVIPT single trials were excluded for a number of different reasons. In most cases where trials were excluded, the number of force pulses was less than 5 (Figure 4A). If participants started too early but there were still 5 force pulses in total, the trial was excluded for the reaction time but not for the other variables (Figure 4B). Trials with more than 5 force pulses and wrong colour sequences were excluded (Figure 4C). Trials with wrong colour sequences were not excluded if there were still 5 detected force pulses in total (Figure 4D). If a participant did not move back to 0 in between two pulses (Figure 4E), trials were kept if the minima between two peaks were below 0.075, i.e., 7.5% MVC. Force peaks with an amplitude below 0.075 were not considered as an attempt to reach a target, i.e. these movements were not counted as a force pulse and therefore ignored (Figure 4F). Trials with double peaks were kept if the minima between two peaks were above 0.075 (Figure 4F).


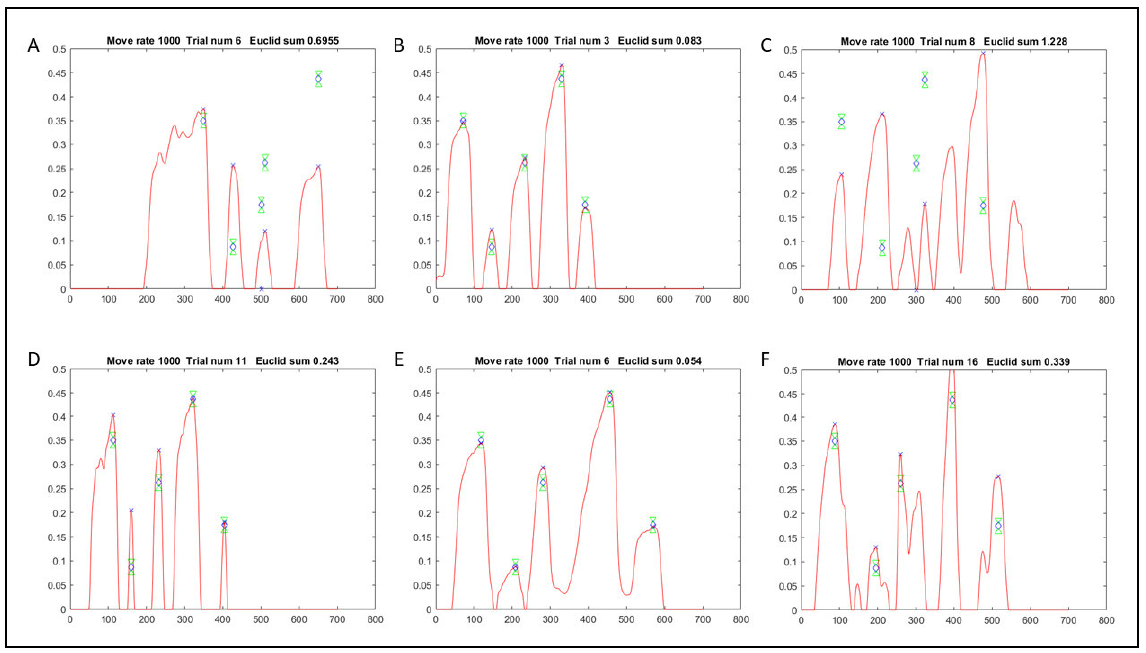


**Supplementary Figure 1.** Example trials. **(A)** Less than 5 pulses (excluded). **(B)** Too early start (only excluded for reaction time). **(C).** More than 5 pulses > 0.075 with wrong targets (excluded) **(D)** Wrong second target but 5 peaks in total (included). **(E)** Minima between two peaks < 0.075 (included). **(F)** Double peaks > 0.075 and one peak < 0.75 (included).
